# Acoustic assessment in mandarin-speaking Parkinson’s disease patients and disease progression monitoring and brain impairment within the speech subsystem

**DOI:** 10.1038/s41531-024-00720-3

**Published:** 2024-06-12

**Authors:** Yu Diao, Hutao Xie, Yanwen Wang, Baotian Zhao, Anchao Yang, Jan Hlavnicka, Jianguo Zhang

**Affiliations:** 1 Department of Neurosurgery, Beijing Tiantan Hospital, Capital Medical University, Beijing, China; 2 Beijing Key Laboratory of Neurostimulation, Beijing, China; 3 Centre of Clinical Neuroscience, 1st Faculty of Medicine, Charles University in Prague, Prague, Czech Republic; 4 Department of Circuit Theory, Faculty of Electrical Engineering, Czech Technical University in Prague, Prague, Czech Republic

**Keywords:** Diagnostic markers, Parkinson's disease

## Abstract

Approximately 90% of Parkinson’s patients (PD) suffer from dysarthria. However, there is currently a lack of research on acoustic measurements and speech impairment patterns among Mandarin-speaking individuals with PD. This study aims to assess the diagnosis and disease monitoring possibility in Mandarin-speaking PD patients through the recommended speech paradigm for non-tonal languages, and to explore the anatomical and functional substrates. We examined total of 160 native Mandarin-speaking Chinese participants consisting of 80 PD patients, 40 healthy controls (HC), and 40 MRI controls. We screened the optimal acoustic metric combination for PD diagnosis. Finally, we used the objective metrics to predict the patient’s motor status using the Naïve Bayes model and analyzed the correlations between cortical thickness, subcortical volumes, functional connectivity, and network properties. Comprehensive acoustic screening based on prosodic, articulation, and phonation abnormalities allows differentiation between HC and PD with an area under the curve of 0.931. Patients with slowed reading exhibited atrophy of the fusiform gyrus (FDR *p* = 0.010, *R* = 0.391), reduced functional connectivity between the fusiform gyrus and motor cortex, and increased nodal local efficiency (NLE) and nodal efficiency (NE) in bilateral pallidum. Patients with prolonged pauses demonstrated atrophy in the left hippocampus, along with decreased NLE and NE. The acoustic assessment in Mandarin proves effective in diagnosis and disease monitoring for Mandarin-speaking PD patients, generalizing standardized acoustic guidelines beyond non-tonal languages. The speech impairment in Mandarin-speaking PD patients not only involves motor aspects of speech but also encompasses the cognitive processes underlying language generation.

## Introduction

Parkinson’s disease (PD) is the second most prevalent neurodegenerative disorder^1,2^. In China, there are currently over 3 million PD patients. It is anticipated that by the year 2030, the number of PD patients in China will reach 5 million, constituting half of the global PD patients^3,4^. Speech disorder in PD called hypokinetic dysarthria has been observed in up to 90% of the PD patients^5,6^. As the most complex quantitative indicator of motor function, speech exhibits remarkable sensitivity to neural damage, making it a compelling and promising biomarker^7,8^. Existing literature on non-tonal language exibited hypokinetic dysarthria characterized by reduced loudness, monotonicity, monoloudness, reduced articulatory precision, and altered speech rate in PD^9,10^. Recent advances in acoustic analysis techniques can provide such an objective feedback with rigorous guidelines^11^.

Various acoustic parameters measured by the state-of-the-art technologies such as the Praat^12^, wavesurfer^13^, Dysarthria Analyzer^14^, and commercial Visi-Pitch^15^ proved to be sensitive to prodromal, early, and developed stages of PD for non-tonal languages^16^. However, one of the main prerequisite for acoustic analyses is that the speech paradigms are suitable for the language given its pronunciation characteristics. Existing research on speech disorders in PD is predominantly conducted in English, accounting for 65% of the studies^17^. Mandarin Chinese is the most widely spoken native language in the world and the predominant language among the Chinese population, yet it belongs to the tonal languages, presenting numerous differences from non-tonal languages commonly used in speech disorders^8,18^. Mandarin Chinese convey both semantic and phonological information through logograms^19^, and defines syllables as a combination of four lexical tones with vowels and consonants demonstrating specific profiles of prosody, pitch variations and regularity^20,21^. Therefore, existing findings and speech protocols as defined in the guidelines^22^ can not be directly generalized without further research. Improving the mentioned acoustic technologies to tonal languages would require a thorough investigation of linguistic relevance, technical implementation, clinical validity, and relation to underlying pathophysiology.

Furthermore, for the dramatic impairment in quality of life and social isolation brought by speech disorders in PD patients, espically for advanced-stage PD patients, current PD therapeutic approaches, such as dopamine-based pharmacotherapy and deep brain stimulation (DBS)^22–24^, fall short in providing substantial benefits. Personalized and objectively monitored interventions are required for the effective management of speech disorders in PD. Exploring the specific brain regions associated with speech disorders lays the groundwork for devising more precise treatment strategies for PD.

The recent calls to increase inclusion of diverse populations in clinical research^7,25–27^ underline the need for comprehensive studies on underrepresented language groups to further our understanding of PD. We aim for the first time to address these issues by progressing on the current clinical guidelines^22^ to bridge the barrier between non-tonal and tonal languages and figuratively the Eastern and Western World in the domain of acoustic analyses of speech in PD. We will investigate the comprehensive acoustic profiles of Mandarin-speaking PD patients on four standard speech paradigms using semi-supervized measurements and compare their relation to motor status and magnetic resonance imaging (MRI) measurements of structure and function of brain.

## Results

### Clinical characteristics of the participants

A total of 160 participants with native Chinese Mandarin language proficiency were recruited for the study. The cohort consisted of 40 healthy controls (18 males, 22 females) with an average age of 60.68 years, 40 MRI controls (21 males, 19 females) with an average age of 59.83 years, and 80 PD patients (45 males, 35 females, please see Table 1 for more details) with an average age of 62.34 years, PD motor symptom duration of 8.61 (SD = 3.83) years, and mean MDS-UPDRS part III score of 45.56 (SD = 17.96). Please see Table 1 for a summary of clinical characteristics.

**Table 1:** Clinical characteristics of PD patients

	PD patients Mean ± SD (range)	Speech control Mean ± SD (range)	MRI control Mean ± SD (range)	*p*
Sex (M/F)	45/35	18/22	21/19	0.513
Age (years)	62.34 ± 8.87 (44–80)	60.68 ± 8.11 (45–77)	59.83 ± 6.14 (51–74)	0.160
Height (cm)	166.26 ± 7.92 (150–185)	164.23 ± 7.10 (150–182)	NA	0.176^a^
Weight (KG)	66.42 ± 11.66 (45–96)	67.70 ± 9.97 (48–88)	NA	0.558^a^
Disease duration	8.61 ± 3.83 (3–25)	NA	NA	-
Smoking status (Y/N)	10/70	6/34	NA	0.778^b^
MDS-UPDRS-III (med off)	45.56 ± 17.96 (10–91)	NA	NA	-
MDS-UPDRS-III (med on)	23.96 ± 13.11 (4–72)	NA	NA	-
MDS-UPDRS-III-Speech (med off)	1.09 ± 0.83 (0–3)	NA	NA	-
MDS-UPDRS-III-speech (med on)	0.55 ± 0.69 (0–2)	NA	NA	-
MoCA	23.73 ± 3.71 (16–30)	25.68 ± 1.27 (24–29)	NA	0.002^a^
Hoehn & Yahr scale	2.86 ± 0.74 (1.5–5)	NA	NA	-
L-dopa equivalent (mg/day)	872.00 ± 347.49 (0–2073)	NA	NA	-
Voice Handicap Index (VHI)	29.25 ± 26.76 (0–100)	NA	NA	-

Baseline data are described using mean and standard deviation, Three-group comparisons were conducted using one-way ANOVA, while comparisons between patient group and Speech control were performed using a two-sample t-test.

^a^Two sample t-test. The inter-group comparison regarding smoking status was conducted using Fisher’s z-test, where Y represents the number of smokers, and N represents the number of non-smokers.

^b^Fisher’s z-test. PD Parkinson’s disease, MDS-UPDRS movement disorder society-unified Parkinson disease rating scale, MoCA Montreal cognitive assessment.

### Speech features

**Fig. 1:**
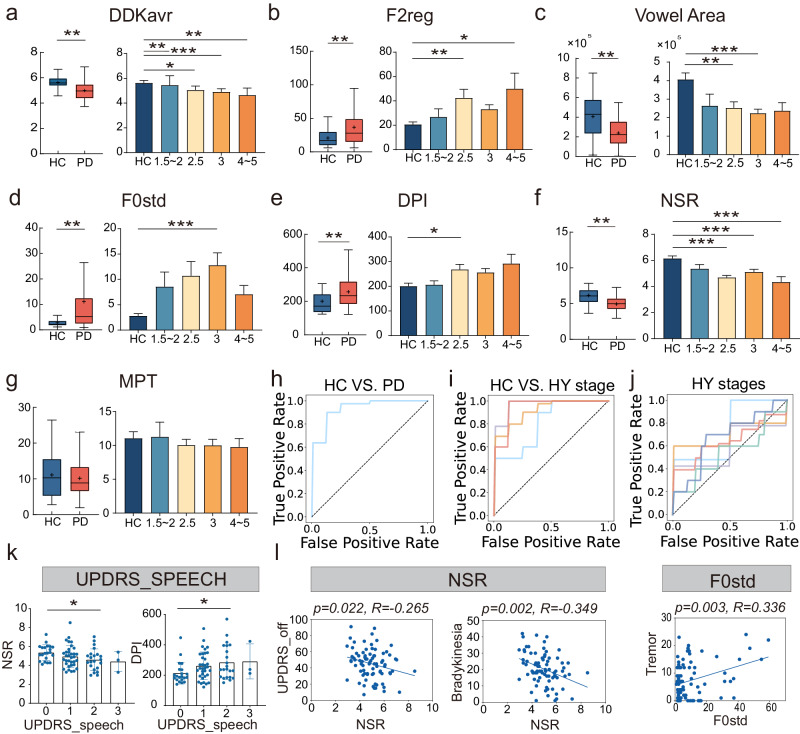
The clinical features of selecting matrices. **a**–**g** Significant differences exist between PD and HC in diadochokinesis, second formant transition, fundamental frequency, vowel space area, as well as pause and speech rate. Two-sample *t*-test was used to analyze HC and PD groups. Statistically significant differences between H-Y PD groups and HC group after Bonferroni adjustment. **h**–**j** The ROC curves were generated by plotting the true positive rate against the false positive rate by AUC with the Naïve Bayes. **i** Blue (HY1.5–2.0 vs HC) with AUC 0.8125, Purple (HY2.5 vs HC) with AUC 0.9725, Orange (HY3.0 vs HC) with AUC 0.9201, Red (HY4–5 vs HC) with AUC 0.9201; **j** Blue (HY2.5 vs 1.5–2.0) with AUC 0.7400, Red (HY2.5 vs 3.0) with AUC 0.6477, Green (HY2.5 vs 4–5) with AUC 0.6477, Purple (HY3.0 vs 1.5–2.0) with AUC 0.5982, Blue (HY3.0 vs 4–5) with AUC 0.5982, Orange (HY4–5 vs 1.5–2.0) with AUC 0.7000. **k**, **l** Prediction of UPDRS scores and sub-UPDRS scores using acoustic metrics through linear regression model and one-way analysis of variance (ANOVA) was conducted to compare with distinct speech scores. The Least Significant Difference (LSD) test was employed for pairwise comparisons between the patient groups. DDKavr average diadochokinesis rate, F0std standard deviation of F0, DPI duration of pause intervals, NSR net syllable rate, F2reg regularity of the second formant variations, MPT maximum phonation time, UPDRS Unified Parkinson’s Disease Rating Scale, PD Parkinson’s disease, HC speech controls. *p < 0.05, **p < 0.01, ***p < 0.001. The error bars represent the standard deviation of the mean.

PD and HC groups were significantly different for a total of 19 speech features. Please see a summary in Table 2. The representative speech characteristics selected for the classification experiment are slow average diadochokinetic rate (DDKavr, *p* < 0.001), slow net speech rate (NSR, *p* < 0.001), longer duration of pause intervals (DPI, *p* = 0.002), increased standard deviation of F0 in sustained vowel /a/ (F0std, *p* < 0.001), increased regularity of the second formant variations (F2reg, *p* < 0.001), and reduction in vowel space area (Vowel Area, *p* < 0.001). The Maximum phonation Time (MPT) did not differ between PD and HC (*p* = 0.417). Furthermore, specific differences in selecting speech characteristics among PD patients at different HY stages are illustrated in Fig. 1. The features are described in more detail in Table 3.

**Table 2:** Disparities in acoustic metrics

	Speech Control		PD			
	mean	SD	mean	SD	*P*	Hedge’s g
Average Diadochokinesis Period (DDKavp)	90.96	12.79	104.28	19.20	<0.001	0.763
Standard Deviation of Diadochokinesis Period (DDKsdp)	35.07	25.70	57.54	38.66	<0.001	0.639
Average Diadochokinetic Rate (DDKavr)	5.63	0.62	4.99	0.77	<0.001	0.875
Coefficient of Variation of Diadochokinesis Period (DDKcvp)	36.44	21.29	51.16	26.87	0.002	0.581
Perturbations of Diadochokinesis Period (DDKjit)	40.20	31.00	65.96	48.18	0.001	0.592
Standard Deviation of DDK Peak Intensity (DDKsdi)	3.56	1.07	4.05	1.21	0.029	0.410
Coefficient of Variation of Diadochokinesis Peak intensity (DDKcvi)	9.94	5.10	13.66	5.29	<0.001	0.707
Voice Onset Time (VOT)	32.45	6.28	33.15	4.62	0.492	0.120
Magnitude of Variations of the Second Formant (F2magn)	1717.01	200.87	1679.77	208.23	0.347	0.180
Rate of the Second Formant Variations (F2rate)	5.22	0.73	4.97	0.71	0.073	0.353
Regularity of the Second Formant Variations (F2reg)	20.70	13.27	36.74	27.83	<0.001	0.664
Average of the Second Formant (F2aver)	1559.27	185.04	1556.49	202.27	0.940	0.014
Minimum of the Second Formant (F2min)	555.70	113.84	598.77	157.78	0.090	0.296
Maximum of the Second Formant (F2max)	2730.40	361.71	2565.24	280.61	0.014	0.530
Fundamental Frequency (F0)	175.62	42.14	160.22	47.09	0.073	0.336
Standard deviation of F0 (F0std)	2.80	1.35	11.09	13.36	<0.001	0.752
Variations of Fundamental Frequency (vF0)	1.63	0.78	7.19	8.95	<0.001	0.752
Standard Deviation of Amplitude (Ampstd)	3.83	1.46	4.18	1.03	0.181	0.291
Variation of Amplitude (vAmp)	11.24	6.28	14.83	5.81	0.003	0.597
Maximum Phonation Time (MPT)	11.08	6.11	10.17	5.09	0.417	0.160
Harmonic to noise ratio (HNR)	18.51	3.72	19.83	4.15	0.082	0.330
Vowel Space Area (VowelArea)	405882.13	222362.83	238256.90	148346.25	<0.001	0.945
Running Mean Fundamental Frequency (rF0)	159.96	30.00	153.12	36.39	0.277	0.197
Running Standard Deviation of F0 (rSTD)	35.20	12.03	30.17	11.80	0.033	0.420
Running Variation of F0 (rvF0)	21.57	4.81	19.41	4.97	0.025	0.436
Running variation of Amplitude (rvAm)	32.19	3.40	30.40	4.33	0.015	0.440
Total duration of Speech (TotalDur)	32.45	5.83	38.19	9.52	<0.001	0.673
Net syllable rate (NSR)	5.13	1.01	4.12	0.92	<0.001	0.903
Duration of pause intervals (DPI)	200.56	78.11	256.90	96.40	<0.001	0.640

A two-sample t-test was used for intergroup analysis, with a significance level of *P* < 0.05 and a two-tailed test.

**Table 3:** Description of Speech Features

Feature	Abbreviation	Paradigm	Description
Average Diadochokinetic Rate (sequence / s)	DDKavr	/Pa/	The average number of /pa/ per second
Average Diadochokinesis Period (ms)	DDKavp	/Pa/	The average duration of each syllable measured at half prominence
Standard Deviation of Diadochokinesis Period (ms)	DDKsdp	/Pa/	The ability of subjects to maintain stable periodic pronunciation, measured in terms of the degree of deviation from DDKavp
Coefficient of Variation of Diadochokinesis Period (%)	DDKcvp	/Pa/	The ratio of DDKstd to DDKavp, measured whether there is a vocalization change in the rate over the 7-s window
Perturbations of Diadochokinesis Period (%)	DDKjit	/Pa/	Another measure of the variability of C-V movements, which is used to assess whether a stable rate can be maintained between cycles of C-V
Standard Deviation of DDK Peak Intensity (dB)	DDKsdi	/Pa/	The standard deviation of DDK peak intensity, used to evaluate the ability of subjects to maintain stable amplitude during phonation
Coefficient of Variation of Diadochokinesis Peak intensity (%)	DDKcvi	/Pa/	The ratio of DDKavi to DDKsdi, used to measure the variability of phonation amplitude
Voice Onset Time (ms)	VOT	/Pa/	The time interval between the release of a stop consonant and the onset of vocal cord vibration, which starts voicing for the following vowel
Magnitude of Variations of the Second Formant (Hz)	F2magn	/i/-/u/	The magnitude of the variations of the second formant during V-V alternation, poorer articulatory control leads to a lower degree of formant change
Rate of the Second Formant Variations (/s)	F2rate	/i/-/u/	The rate of alternation in the second formant
Regularity of the Second Formant Variations (%)	F2reg	/i/-/u/	The regularity of the second formant, correlated with the degree of alternation regularity in articulatory movement, with lower values indicating greater regularity
Average of the Second Formant (Hz)	F2aver	/i/-/u/	The average F2 value for the vocalization
Minimum of the Second Formant (Hz)	F2min	/i/-/u/	The lowest F2 value for the vocalization
Maximum of the Second Formant (Hz)	F2max	/i/-/u/	The highest F2 value for the vocalization
Fundamental Frequency (Hz)	F0	/a/	The average F0 of the subjects throughout the pronunciation process of /a/
Standard deviation of F0 (Hz)	F0std	/a/	The degree of deviation from F0
Variations of Fundamental Frequency (%)	vF0	/a/	The ratio of the standard deviation of F0 to the mean of F0
Standard Deviation of Amplitude (dB)	Ampstd	/a/	The degree of deviation from Amp
Variation of Amplitude (%)	vAmp	/a/	The ratio of the standard deviation of Amp to the mean of Amp
Maximum Phonation Time (s)	MPT	/a/	The maximum duration of the pronunciation of /a/
Harmonic to noise ratio (dB)	HNR	/a/	The ratio of harmonic energy to non-harmonic (noise) energy in the signal
Vowel Space Area (Hz^2^)	VowelArea	/a/, /i/, and /u/	The vowel space area determined by the first and second formants of the vowels /a/, /i/, and /u/
Running Mean Fundamental Frequency (Hz)	rF0	Reading passage	The average F0 of the subjects throughout the paragraph
Running Standard Deviation of F0 (Hz)	rSTD	Reading passage	The standard deviation of rF0, illuminated the change of pitch in tonality that occur during the act of reading
Running Variation of F0 (%)	rvF0	Reading passage	The ratio of the standard deviation of rF0 to the mean of rF0 during paragraph reading
Running variation of Amplitude (%)	rvAm	Reading passage	The ratio of the standard deviation of amplitude to the mean of amplitude during paragraph reading
Net syllable rate (syllable/s)	NSR	Reading passage	The number of syllables (i.e., Chinese characters) per second divided by the net speech time.
Total duration of Speech (s)	TotalDur	Reading passage	The total time required for paragraph reading
Duration of pause intervals (ms)	DPI	Reading passage	The median duration of pauses.

The Naïve Bayes model combining DDKavr, NSR, DPI, F0std, F2reg, and VowelArea showed an overall AUC value of 0.931 for distinguishing between PD and HC. Additionally, the AUC for discriminating between HC and different HY stages of PD is depicted in Fig. 1h.

### Relationship of speech features and clinical variables

As age, gender, MoCA, height, and weight are covariates, the passage reading metrics NSR (*p* = 0.002, *R* = −0.349) were significantly associated with bradykinesia (Med-Off) and MDS-UPDRS III (Med-Off) (*p* = 0.022, *R* = −0.265). Additionally, F0std was negatively correlated with tremor (Med-Off) (*p* = 0.003, *R* = 0.336). Furthermore, when comparing the speech subscores of MDS-UPDRS III between groups based on the “Med-Off” state, we observed significant differences in DPI and NSR across different speech ratings. Correlations are plotted in Fig. 1i, j.

### Relationships between acoustic measurements and anatomical structure

**Fig. 2:**
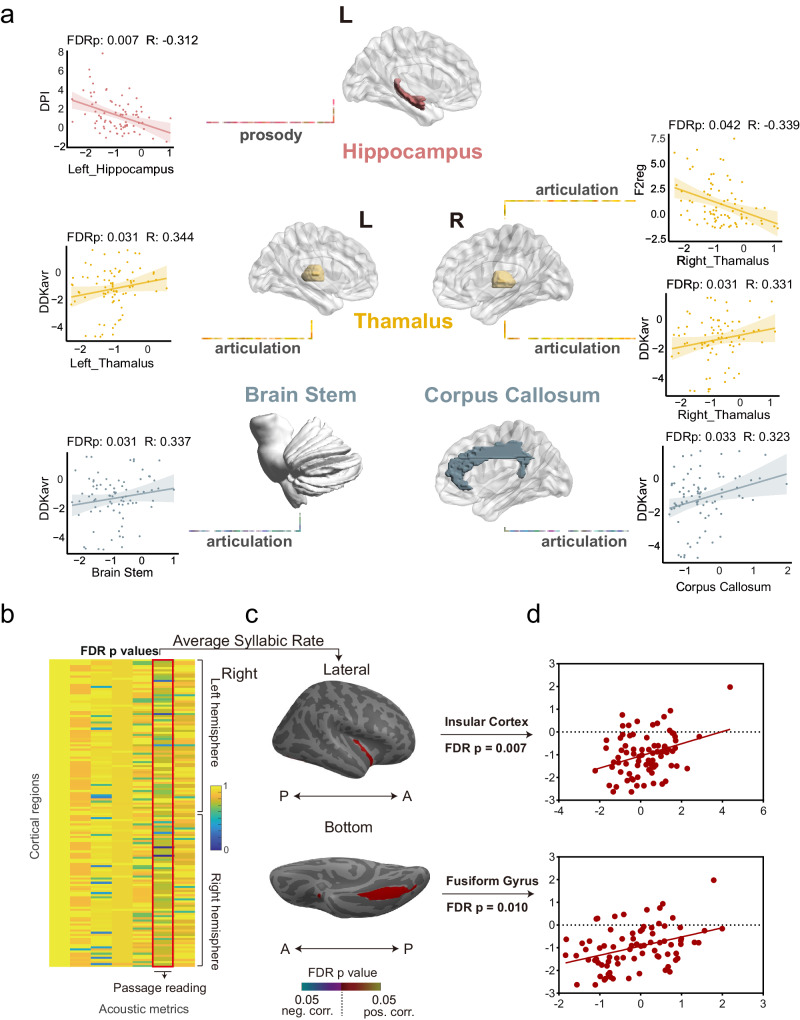
The overall partial correlation results between Destrieux atlas-based cortical thickness and acoustic metrics after regressing out covariates in PD patients. **a** the overall correlation results between Destrieux atlas-based volume of subcortical nuclei and acoustic metrics. **b** The FDR *p* values distribution of the partial correlation between normalized cortical thickness and acoustic metrics. Most of the significant correlations were observed within the passage reading paradigm related measurements. **c** The FDR *p* values were projected to the corresponding anatomical regions, and brain regions that survived the FDR *p* < 0.05. The detailed areas and statistics were: Right: lateral fusiform gyrus (FDR *p*: 0.010 *R*: 0.391) and giant Insula and superior central sulcus (FDR *p*: 0.007 *R*: 0.405). **d** Linear regression plot of cortical thickness of brain regions after FDR correction, with respect to NSR. FDR false discovery rate, A anterior, P posterior, neg. negative, pos. positive, corr. correlation. DDKavr average diadochokinesis rate, F2reg regularity of the second formant variations, DPI duration of pause intervals, NSR net syllable rate.

With atlas-level, after regressing out the age, gender, MoCA score, height, weight, and dominant hand as covariates, slow reading measured by NSR is positively correlated with the thickness of the right lateral fusiform gyrus (FDR *p* = 0.010, *R* = 0.391) and right insula (FDR *p* = 0.007, *R* = 0.405). DPI in the reading passage is negatively correlated with the left hippocampus (FDR *p* = 0.071, *R* = −0.312) (Fig. 2b).

The DDKavr in diadochokinesis is positively correlated with the left thalamus (FDR *p* = 0.031, *R* = 0.344), right thalamus (FDR *p* = 0.031, *R* = 0.331), brainstem (FDR *p* = 0.031, *R* = 0.337), and corpus callosum (FDR *p* = 0.033, *R* = 0.323).

F2reg measured in /i/-/u/ repetition paradigm is negatively correlated with the right thalamus (FDR *p* = 0.042, *R* = −0.339). The results are visualized in Fig. 2. Further vertex-wise correlation replicating similar results as shown in the Supplementary Table 2.

### Relationships between acoustic measurements and functional connectivity and network properties

At the atlas level, after regressing out the age, gender, MoCA score, height, weight, and dominant hand as covariates, there was a significant negative correlation between whole-brain aCp and F0std (*p* = 0.031, *R* = −0.680), while a significant positive correlation was observed between whole-brain aEloc and DDKavr (*p* = 0.037, *R* = 0.663).

**Fig. 3:**
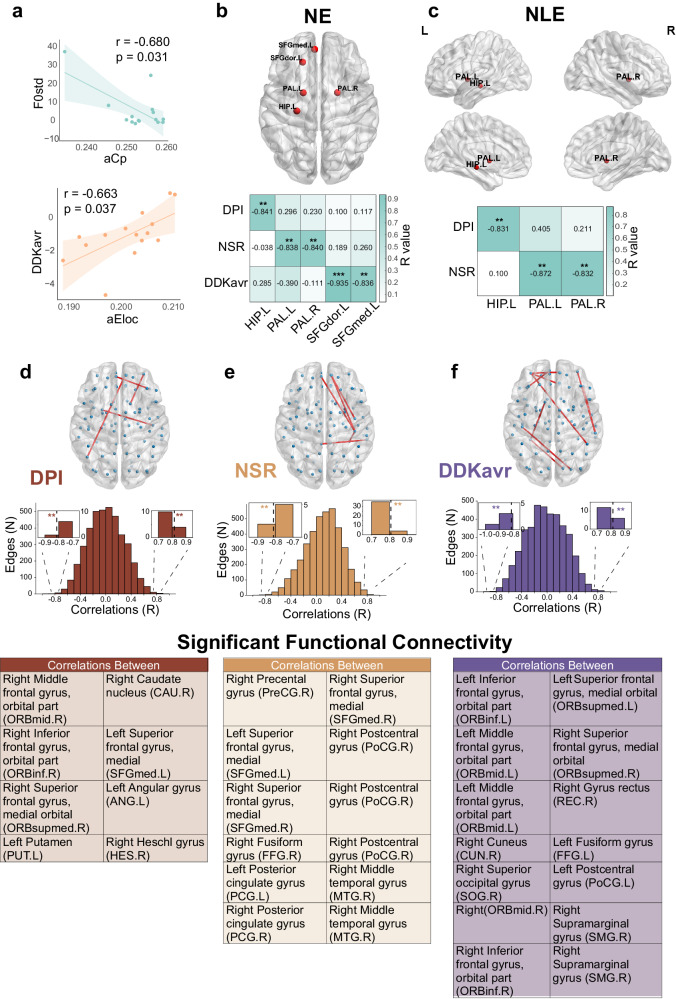
Relationships between acoustic measurements and functional connectivity and network properties after regressing out covariates in PD patients. **a** Linear regression plots depicting the associations between speech metrics and global properties after covariate regression. **b**, **c** Correlations between nodal local efficiency, nodal efficiency, and speech metrics after covariate regression (*p* < 0.005, uncorrected). **d**–**f** Correlations between language metrics at the AAL90 atlas level and functional connectivity (*p* < 0.005, uncorrected) after covariate regression. DDKavr average diadochokinesis rate, DPI duration of pause intervals, NSR net syllable rate. aCp average clustering coefficient, aEloc average network efficiency, NLE nodal local efficiency, NE nodal efficiency, HIP hippocampus, PAL pallidus, SFGdor superior frontal gyrus, dorsal, SFGmed superior frontal gyrus, medial.

Regarding node efficiency, DPI exhibited a negative correlation with left hippocampus (uncorrected *p* = 0.002, *R* = −0.841), NSR showed negative correlations with left pallidum (uncorrected *p* = 0.003, *R* = −0.838) and right pallidum (uncorrected *p* = 0.002, *R* = −0.840). DDKavr displayed negative correlations with left dorsolateral superior frontal gyrus (uncorrected *p* < 0.001, *R* = −0.935) and left medial superior frontal gyrus (uncorrected *p* = 0.003, *R* = −0.836) (Fig. 3b). For node local efficiency, DPI was negatively correlated with left hippocampus (uncorrected *p* = 0.003, *R* = −0.831), NSR showed negative correlations with left pallidum (uncorrected *p* = 0.001, *R* = −0.872) and right pallidum (uncorrected *p* = 0.003, *R* = −0.832) (Fig. 3c).

DPI exhibited positive correlations with FC of right middle frontal gyrus and right caudate, FC of right inferior frontal gyrus and left superior frontal gyrus, FC of right superior frontal gyrus and left angular gyrus; and negative correlation with FC of left putamen and right Heschl’s gyrus. NSR showed positive correlations with FC of right precentral gyrus and right superior frontal gyrus, FC of left, right superior frontal gyrus, right fusiform gyrus and right postcentral gyrus; negative correlations with FC of left posterior cingulate gyrus, right posterior cingulate gyrus, and right middle temporal gyrus. The FC results of DDK, along with correlation coefficients, are detailed in Fig. 3 and Supplementary Table 4.

## Discussion

This study for the first time extends of standard clinical guidelines^22^ for tonal languages, highlighting the effectiveness of a comprehensive acoustic analysis of speech for Chinese-speaking patients with PD and conducting MRI studies on their potential brain functions and structures. We observed the possibility of acoustic features combination in Mandarin for diagnosis and disease monitoring in PD. Additionally, localized atrophy in the hippocampus and fusiform gyrus, reduced nodal efficiency in the hippocampus and pallidum, and decreased functional connectivity between the cingulate gyrus, motor cortex, and inferior frontal gyrus, were correlated with the speech production and reading.

The speech disorder characteristics observed in Chinese-speaking PD cohort were partially different to those in atonal languages^5,6,8–10,28^. Due to differences between Chinese and Indo-European languages, the presence of four tones may render Running Standard Deviation of F0 (rSTD) and Running Variation of Amplitude (rvAm) during reading less sensitive to distinguishing abnormal speech states in PD. However, many studies in non-tonal languages have identified differences of rSTD and rvAm between PD and HC^7,8^. We attribute this to the dominance of tones in expressing meaning during Chinese reading, as opposed to emphasis on stress and F0 fluctuations. This suggests that the four tones in Chinese-speaking PD patients may not be significantly impaired, but further research centered on tonal changes is required for confirmation.

Although we did not include non-tonal languages in this study, we followed comparable examination protocols and used identical analytical software for the calculation of our key metrics of voice of onset (VOT), NSR, and DPI as in the previous multicentric study covering Czech, German, English, French, and Italian^8^. The result on VOT in Chinese did not reveal significant differences, which can be attributed to the distinct pronunciation of consonants in English and the Mandarin Pinyin initials, when measuring VOT during /pa/ pronunciation, the Chinese /pa/ sound has a longer VOT compared to non-tonal languages^29^. Nevertheless, vowel pronunciation in Chinese and Indo-European languages exhibit similarity, with impaired articulation reflected in the reduction of vowel area among PD patients^11^. Furthermore, despite significant differences in stress, grammatical structure, and vocabulary systems between Chinese and Indo-European languages, NSR and DPI show comparable trends and effectivity in differentiating PD and HC^8,11,30^. Previous studies have also highlighted differences between Chinese and Polish, emphasizing that vocal features sensitive to diagnosis in non-tonal languages may not be effective in Chinese^31^. This underscores the importance of screening language features specific to Chinese.

The vast majority of selected acoustic features showed significant group differences between HC and PD and these differences persist even after stratifying by H-Y stage. The combination of six distinct features offered high performance (AUC > 0.9) in PD diagnosis. Additionally, while the model demonstrates only moderate performance in classifying HY stages, the relationships between NSR and bradykinesia, as well as F0std and tremor, indicate that vocal biomarkers are associated with disease progression. In comparison to the study conducted by ref. ^32^, who aimed to build the high performance model from a small dataset (*N* = 34) from technique perspective, we provided further insight on the clinical interpretability while keeping a comparable AUC over 0.9 in a larger dataset. Such approach could be of great benefit for remote assessment and disease monitoring for the Chinese PD population^33^. Furthermore, the preoperative speech disorders are one of the crucial factors influencing the long-term speech prognosis of patients after DBS^34^ and it is equally essential to evaluate the speech disturbance utilizing appropriate paradigms and speech metrics during DBS surgery^35^. Hence, quantitative acoustic evaluation in Mandarin also holds significant importance for DBS candidates. Notably, the foundation of this study lies in the methodologies developed by Jan Rusz’s lab^14,36,37^ and the MSP Program. Although the proposed speech analyses were newly adapted to the Mandarin tonal language and thus were performed under supervision to achieve full control over the quality of the processing, we hypothesize that the final solution can be computerized since the Dysarthria Analyzer^14^ as well as the MSP Program are already fully automated.

In addition to all the possible benefits, the compelling one is the discovery of relationships between vocal biomarkers and brain structures and network. Impairment in the reading passage of PD patients may not only involve damage to articulation or prosody but also extend to higher-order language deficits^38^. The correlation between hippocampus atrophy, local efficiency impairment, and DPI highlights the role of the hippocampus in language processing^39^. The hippocampus is implicated in cognitive language processing, including semantic encoding^40,41^. It combines incoming words with stored semantics^39^, engages in sensory prediction based on associative contextual representations encoded in the hippocampus, and ultimately provides feedback by coupling with the auditory cortex, thus “predicting” the expression of sentences^42^. The observed reduction in functional connectivity between the temporal lobe and the striatum further supports this notion. Additionally, it is noteworthy that Mandarin-speaking patients encode sentences by syllables and express semantics through word phrases^43^. The relationship between DPI and the hippocampus further underscores the connection between pauses and semantic expression in Mandarin^44,45^. This further underscores that the mid-to-late stages of PD patients experienced a spectrum of mixed speech disorders involving both cognitive and motor control levels.

Additionally, reduced activation is noted in the premotor cortex in PD patients experiencing speech difficulties, aligning with our findings of decreased functional connectivity between the right precentral gyrus and the right medial superior frontal gyrus in patients with slowed speech^46^. Consistent with our results, a recent study also reported an association between reading impairment and damage to the cingulate gyrus in PD patients^47^. The dual-pathway theory of reading attributes the SFG and fusiform to “access to meaning,” while assigning the precentral gyrus to “access to pronunciation and articulation”^48^. This corroborates the atrophy of the fusiform gyrus, along with reduced functional connectivity observed in the fusiform gyrus, superior frontal gyrus, and precentral gyrus in PD patients with slowed NSR, suggesting potential impairments in speech encoding and motor generation functions^37,49^. In addition to cortical regions, PD patients with speech disorders exhibit aberrant functional connectivity between the globus pallidus and premotor cortex^50^, we similarly observed compensatory increases in nodal efficiency and local efficiency of the pallidus in PD patients with slowed speech. This suggested the damage to speech in PD may entail a broad-scale network disruption encompassing both cortical and subcortical regions^50,51^. The reduced overall efficiency further supports this notion.

Thalamus is significantly involved in speech perception and production^52,53^ as underlined by correlation between atrophy of thalamus and articulation. In previous fMRI studies of DBS candidates, we identified changes consistent with our functional connectivity results. Furthermore, our study revealed a correlation between these abnormal functional connections and speech metrics^54^. Articulation disorders may be more closely related to brain regions associated with motor control, such as the thalamus, while prosody disorders may be more connected to the entire process of speech production. Specific vocal biomarkers are key for the functional localization of lesion following the landmark work by ref. ^55^ who established the hypothesis and linked structural damage to motor speech disorders. The presented findings could help not only with the study of basal ganglia and diagnosis but also with personalization of neuromodulation therapies and experimental design of invasive intracranial recordings^53,56^.

Only Mandarin-speaking candidates with a disease duration of 5 years or more were included in the PD cohort, which limits the generalizability of our results to tonal languages. Additionally, the brain functional correlations were exploratory and not subject to rigorous multiple comparison correction. Studies on brain structure and function are predominantly observational, and have less of fMRI data, and future work should employ intervention approaches such as transcranial stimulation to validate the association of specific brain areas to speech manifestations.

To summarize, we identified a tremendous diagnostic value of the proposed comprehensive acoustic evaluation of Mandarin speech for the rapid determination of PD progression stages as well as localization within different speech subsystems of structural and functional impairments in the hippocampus, cingulate gyrus, and basal ganglia. This study extends the conventional speech examination protocols and paradigms based on non-tonal languages^22^, which creates an opportunity for the vast language group to share harmonized results, contributing to the expansion of our knowledge about PD.

## Methods

### Study design and participants

**Fig. 4:**
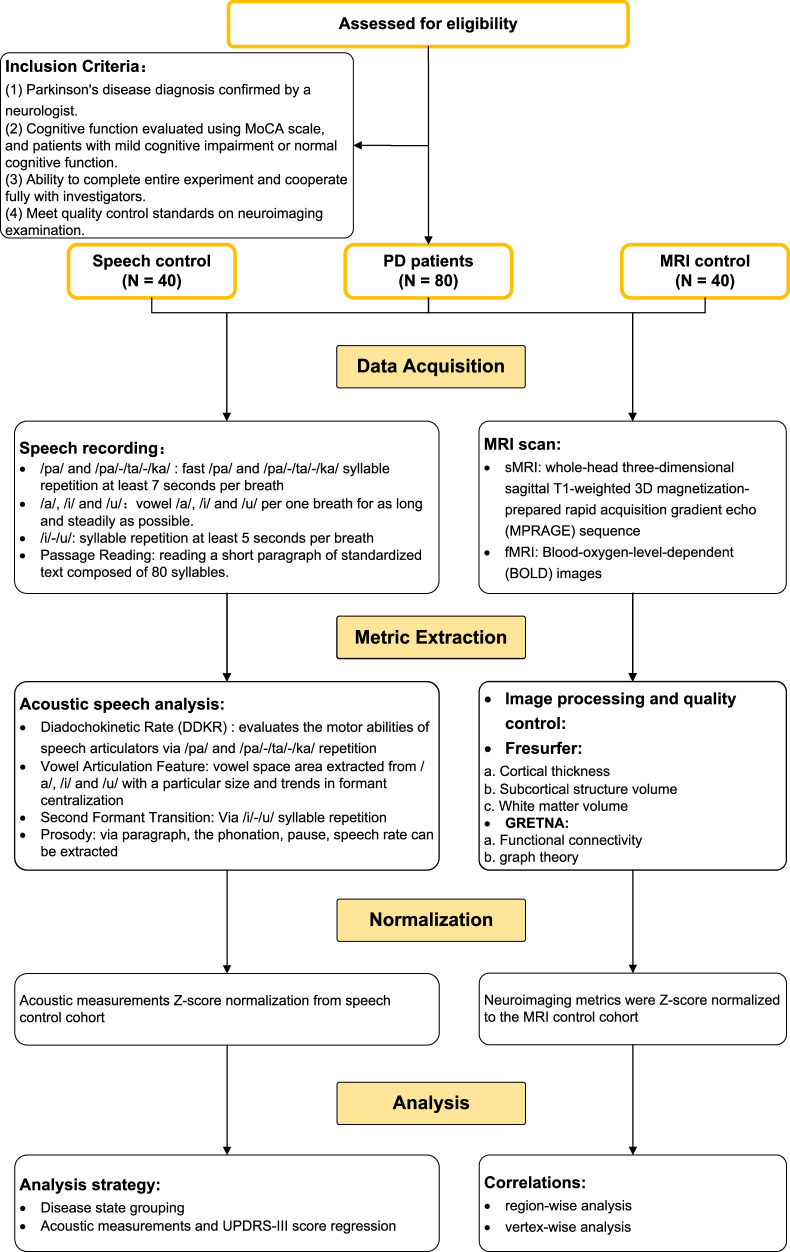
Participant flowchart. After applying inclusion and exclusion criteria, we included PD patients and normal subjects. Speech recordings were obtained from PD and speech control groups using recording equipment for subsequent speech analysis. FreeSurfer and GRETNA were used to process MRI scans, obtaining both structural and functional MRI data for PD patients and MRI controls. Data analysis was conducted after normalization based on the control groups.

Total of 80 PD patients, 40 healthy controls (HC) for speech examination, and 40 normal MRI controls were prospectively recruited from October 2022 to April 2023 at Beijing Tiantan Hospital, Capital Medical University, China. The inclusion criteria for PD cohort were: (1) the diagnosis of idiopathic PD and meeting the UK Brain Bank criteria for PD^57^; and with a disease duration of 5 years or more; (2) normal cognitive function (scores of Montreal Cognitive Assessment (MoCA) ≥ 24) or mild cognitive impairment (meeting the criteria for the diagnosis of PD-MCI level I^58^ and 16 ≦ MoCA < 24)^59^; (3) completing the entire experiment and cooperating fully with the investigators; (4) meeting the quality control standards on neuroimaging and speech examination. The severity of motor symptoms was defined using the Movement Disorders Society-Unified Parkinson’s Disease Rating Scale, Parts III (MDS-UPDRS-III) and Hoehn & Yahr scale (H-Y scale), while speech examination was conducted in the medication off-state with at least 8 h after dopamine withdrawal. The healthy controls were selected from healthy individuals without any history of communication disorders or respiratory diseases to match the PD patients age range between 40 and 80 years. The research flowchart and baseline information are in Fig. 4 and Table 1. Ethical approval for this study was obtained from the Beijing Tiantan Hospital, and all patients provided written informed consent, adhering to the principles of the Declaration of Helsinki.

### Clinical examinations

The clinical evaluation of each subject consisted of: (1) a review of their personal and medical history, including information on gender, age, height, weight, and past medical conditions; (2) quantitative testing of motor symptoms of PD using the MDS-UPDRS III; and (3) cognitive testing using the MoCA. All clinical scales and diagnoses were conducted by a neurologist with the expertise in movement disorders.

### Speech examination

**Fig. 5:**
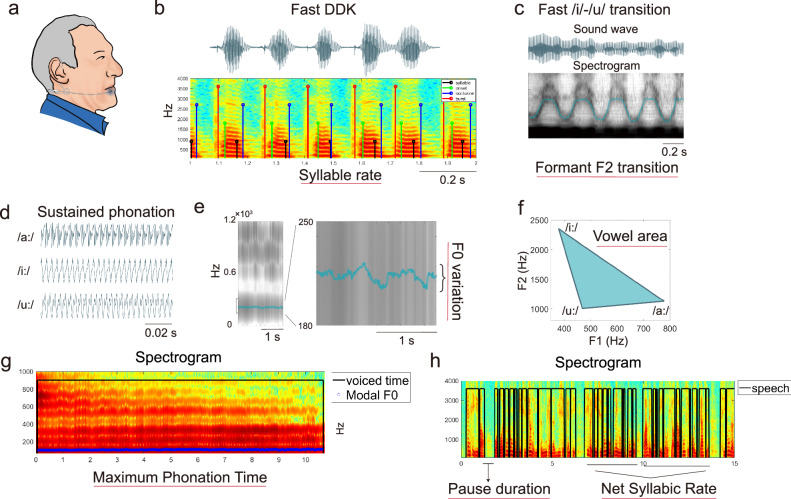
Overview of applied acoustic measurements. **a** The voice was recorded using a head-mounted condenser microphone positioned 2 cm away from the lips. **b** A sound wave example of the DDK paradigm was obtained to measure the speed related indicators of the DDK alternate motion. **c** A sound wave example of the fast /i/-/u/ transition was obtained to measure the formant F2 transition related indicators. **d** Sounf waveform examples of monophthong pronunciation was used to obtain the first and second formant related indicators for each vowel sound. **e** The sound waveform example of the pronunciation of /a/ was used to obtain relevant indicators such as fundamental frequency and its variability during the production of a monophthong. **f** The vowel triangle area was obtained by measuring the first and second formant of /a/, /i/, and /u/. **g** An example spectrogram of the vowel /a/ showing the maximum phonation time. **h** An example of paragraph reading paradigm was used to obtain relevant indicators such as reading speed and pauses.

The speech was recorded in a closed room with low ambient noise and reverberation levels using a head-mounted condenser omnidirectional microphone (Sennheiser HSP-2, Germany) placed ~2 cm from the participant’s mouth (Fig. 5a). The sampling frequency was set to 48 kHz with 16-bit resolution. Each participant was examined by a trained speech specialist within a single session. Participants were instructed to perform the following vocal tasks with two repetitions: (1) sustained phonation of the vowel /a/, /i/, and /u/ per single breath for as long and steadily as possible; (2) fast diadochokinetic (DDK) syllable repetition at least 7 s per single breath; (3) fast /i/-/u/ syllable repetition at least 5 s per single breath; (4) reading a short passage of a weather report containing 105 Chinese characters (syllables).

We tested both fast single /pa/ and triple syllable /pa/-/ta/-/ka/ for the DDK paradigm in all patients to evaluate the discriminatory effect of syllable types. The effect size (HC vs PD) doesn’t differ significantly between the tasks (Supplementary Table 1), therefore, only the fast /pa/ paradigm was included in the further analysis for simplicity. The paradigms were customized for Mandarin Chinese.

### Parameter extraction

We performed the acoustic analysis using vocal biomarkers carefully selected from established features documented in previous studies on tonal languages to ensure enhanced applicability and comparability with tonal languages.

The audio quality control and trimming were performed in WaveSurfer (https://sourceforge.net/projects/wavesurfer/). We implemented a battery of acoustic features using WaveSurfer and combined with in-house MATLAB scripts and Dysarthria Analyzer (https://www.dysan.cz/)^14^ in a semi-supervised manner in order to gain a more increased and controlled quality of measurements.

The DDK was analyzed by calculating the waveform envelope and applying the *findpeaks* function to identify the syllable onset, syllable nuclei, and syllable offset with manually set constraints such as the threshold of peak’s prominence to get optimal detection accuracy. We described the detected events via standard parameters. Furthermore, the VOT was measured by Dysarthria Analyzer supervised by inspecting the results via spectrogram (more details in Table 3).

The formant estimation in /i/-/u/ repetition paradigm was carried out via linear predictive coding in WaveSurfer with default settings. The transitions of the second formant (F2) were identified with the supervised method similar to the DDK analysis and parameterized with common descriptors (Table 3).

The pitch and amplitude of the sustained vowel /a/ were analyzed using WaveSurfer as follows. We optimized the setting of the pitch detection constraints manually for each recording and calculated the statistics such as mean and standard deviation over the detected F0 sequence in MATLAB. Additionally, we parameterized also amplitude within the F0 intervals by standard deviation and other metrics outlined in Table 3. MPT and Harmonic-to-noise ratio were calculated using the Dysarthria Analyzer^14^ supervised by plotting the results against the spectrogram as illustrated in Fig. 5.

Pauses in the reading passage task were determined as unvoiced and non-consonant signals, including the intervals of respiration following the established criteria^60^. Pauses were identified using an automated method incorporated within the Dysarthria Analyzer^14^, with the minimum pause duration set at 30 ms^60^. All results were supervised by inspecting the results via spectrogram. The net syllable rate (NSR) was calculated as a ratio of 105 syllables read and total net time of reading excluding pause intervals according to definitions in Table 3.

A total of 31 speech parameters were calculated in MATLAB according to definitions summarized in Table 3 based on well-established methods^11,22,61^ and/or inspired by the Motor Speech Profile (MSP) Program (PENTAX Medical, New Jersey, USA) and the Dysarthria Analyzer^14^.

Please see Fig. 5 illustrating speech feature extraction on examples of sound waves for various speech paradigms. The Supplementary Fig. 1 illustrates the semi-supervised measurement process. Please see Supplementary Material 1 and Table 3 for feature details.

### Features selection and normalization

We conducted feature selection to reduce the number of analyzed features and prevent overfitting in the machine learning experiment, while also aiming to provide clinically relevant metrics for practical results interpretation. We selected representative measurements from four speech dimensions including prosody, articulation, phonation, and respiration based on the following criteria: high effect size, high clinical interpretability, and low intraclass correlation. The MPT was included as the key characteristic of respiration to make the final analysis more comprehensive. To enhance comparability with non-tonal languages, we selected the metrics recommended by the clinical guidelines that were previously employed in studies involving PD in non-tonal languages^8,11^. All the measurements of all the participants were normalized to z-scores separately for each gender using the data of healthy controls for further statistical analysis.

### MRI acquisition

Magnetic resonance imaging images of 80 PD patients and 40 MRI controls were acquired using 3.0T MRI scanner (Simens Medical Systems) with 32-channel head coil. A whole-head three-dimensional sagittal T1-weighted 3D magnetization-prepared rapid acquisition gradient echo (MPRAGE) sequence was used (repetition time, 1.56 ms; echo time, 0.00169 ms; flip angle, 8°; matrix size, 256 × 256; isotropic voxel, 1 × 1 × 1 mm^3^; the number of slices, 196).

We included fMRI scans of 16 PD patients with an off-medication period of 12 h or longer. Patients whose head motion exceeded 3 mm of translation were excluded. During the scan, patients were instructed to keep their eyes closed and remain awake. Blood-oxygen-level-dependent images were acquired employing an echo-planar imaging sequence with the following parameters: a repetition time (TR) of 750 ms, an echo time (TE) of 35 ms, a flip angle of 52°, an acquisition matrix of 92 × 92, a field of view (FOV) of 100 mm × 100 mm, a Multiband Acceleration Factor of 8, and a scanning time approximately equal to 600 s.

### Imaging processing

We converted all DICOM files to NifTi format using the SPM12 software (https://www.fil.ion.ucl.ac.uk/spm/software/spm12/) and carefully inspected the data for quality. For data preprocessing and image analysis, we employed the FreeSurfer software (version development, http://www.freesurfer.net), and GRETNA 2.0.0 toolbox (https://www.nitrc.org/projects/gretna/) as previously described^62,63^.

The feature extraction of cortical thickness and subcortical nuclei volume was performed in both vertex-level and atlas-level approaches. We utilized the Destrieux atlas^62^ for cortical segmentation, extracting and calculating the average cortical thickness for each region as the regional thickness. To normalize the regional thickness in each patient, we first normalized the regional cortical thickness by the global mean of cortical thickness at an individual level and subsequently, the results were z-score normalized to the healthy controls^64^. The z-score values of cortical thickness were used in the correlation analysis with acoustic measurements. Similarly, we treated the volumes of subcortical structures (extracted from aseg.stats output file) as subcortical nuclei features, and the normalization was calculated similarly as for the thickness.

The intrinsic connectivity network within the brain is comprised of nodes and edges. Utilizing the AAL90 atlas, the brain is segmented into 90 nodes, and a functional connectivity network (FCN) of size 90 * 90 is constructed by computing correlations of the time series for these 90 nodes. To correct for the non-normality of the correlation coefficients, Fisher’s z transformation was applied to calculate the z-scores. Global properties (average clustering coefficient (aCp) and average network efficiency (aEloc)) and node properties (nodal local efficiency (NLE) and nodal efficiency (NE)) are calculated based on the FCN using graph theory.

### Statistical analysis

Group level feature comparison was conducted using two-sample t-test. The comparison between different HY stages of PD and HC was conducted using one-way ANOVA analysis, with Bonferroni correction applied for multiple comparisons. Age, gender, MoCA, height, and weight were used as covariates for the partial correlations analysis between acoustic features and MDS-UPDRS III.

Additionally, we performed a classification experiment using Gaussian Naïve Bayes classification in a 5-fold cross-validation scheme to differentiate between HC and PD using the set of six selected speech features. The performance was described with the receiver operating characteristic (ROC) and the area under the ROC curve (AUC).

We accounted for the age, gender, MoCA score, height, weight, and dominant hand as covariates. The correlation between standardized speech metrics and standardized cortical thickness/subcortical volume, functional connectivity, and network properties was analyzed using Pearson correlation. In the statistical analysis of structural MRI, the false discovery rate (FDR) correction was applied to control for multiple comparisons across different regions. For fMRI statistical results, given the relatively limited number of patients included and the exploratory design, results with *p*-values below 0.005 are presented without correction.

All Hypotheses were considered two-tailed and tested with the significance threshold of *p* < 0.05. Statistical analyses and visualizations were performed using MATLAB 2021b (MathWorks Inc., Natick, Massachusetts, USA).

### Reporting summary

Further information on research design is available in the Nature Research Reporting Summary linked to this article.

### Supplementary information

Supplement

reporting summary

## Data Availability

The data that support the findings of this study are available from the corresponding author upon request.
