# Supplementary material for: Acoustic assessment in mandarin-speaking Parkinson’s disease patients and disease progression monitoring and brain impairment within the speech subsystem
Source: NPJ Parkinsons Dis. 2024 Jun 12;10:115. doi: 10.1038/s41531-024-00720-3 (PMC11169641; doi:10.1038/s41531-024-00720-3)
Supplement: Supplementary file 1 — Supplement [file 41531_2024_720_MOESM1_ESM.pdf]

Supplementary Material 1

DDK related features

In DDK evaluation, we employed /pa/ repetition to assess sequential motion rate and variation [10, 26]. Specifically, we calculated the average number of /pa/ per second (average DDK rate, DDKavr), the average fundamental frequency of the client during vocalization (average DDK period, DDKavp) and the variability of sequential motion. In addition, the intensity of pronunciation was also measured. We evaluated the variability of speech loudness and the voice onset time (VOT).

Second Formant (F2) Transition

Furthermore, we evaluated the capability of vowel-to-vowel transition in terms of the second formant transition by utilizing the combination of /i/ and /u/ sounds. As these two vowels have different second formant locations, participants were required to modify their articulation to produce the target sounds. This approach provides a more accurate assessment of the movement of the lips and tongue during speech production [51]. Specifically, we calculated six assessment metrics, including magnitude of variations of F2 during vocalization (F2magn), the rate of change (F2rate), regularity of F2 variations (F2reg), the mean values of F2 (F2aver), maximum values of F2 (F2max) and minimum values of F2 (F2min).

F0 and amplitude of sustained phonation

The F0, measured in Hertz (Hz), is believed to reflect the auditory perception of vocal pitch. Amplitude can reflect the degree of auditory perception of sound loudness. Prolonged pronunciation of a single vowel /a/ can effectively reflect the fundamental frequency and amplitude characteristics of PD patients' speech motor function and their degree of variation [52, 53]. We extracted six assessment metrics, including fundamental frequency (F0), average amplitude (Amp), standard deviation of F0 (F0std), variations of F0 (vF0), the standard deviation of amplitude (Ampstd) and the variation of amplitude (vAmp). Additionally, Harmonic to noise ratio (HNR) has been calculated, and to assess respiratory, we also conducted calculations for the maximum phonation time (MPT) of the vowel /a/.

Vowel space area (VowelArea)

The vowel space area (measured in square Hz) is a vowel measurement method that evaluates the working space of the vocal organs by analyzing the first (F1) and second (F2) formants to determine the acoustic characteristics of each vowel pronunciation. Previous studies have shown that

the F1 is correlated with tongue height, while the F2 is correlated with tongue advancement during vowel production [54]. Therefore, we designed a vowel triangle area based on F1 and F2 values of the vowels /a/, /i/, and /u/ according to previous literature [13].

Passage reading

Natural variations in pitch and volume during normal dynamic speech can be extracted by analyzing passage readings. We extracted a total of four assessment metrics related to F0, volume, and their variability from passage reading. We extracted the overall mean F0 (rF0). Additionally, the pitch variability of sarcastic intonations was measured by the standard deviation (rSTD) and variation of F0 (rvF0). Variations in volume that reflect normal speech processes were obtained by analyzing the amplitude variation (rvAm). Aside from that, passage readings can also provide indicators of articulation rate and pause characteristics that are related to the speech motor execution and cognitive linguistic processing. We extracted metrics related to speech rate and pause.

Supplementary Table 1:

	Pa				*Pa-Ta-Ka*			
	meanHC_Pa	meanPD_Pa	P	Hedge's g	meanHC_PaTaKa	meanPD_PaTaKa	P	Hedge's g
DDKavp	90.96	104.28	<0.001	0.763	103.92	120.86	<0.001	0.798
DDKsdp	35.07	57.54	<0.001	0.639	55.38	63.56	0.094	0.312
DDKavr	5.63	4.99	<0.001	0.875	5.13	4.28	<0.001	1.135
DDKcvp	36.44	51.16	0.002	0.581	52.42	51.27	0.735	0.066
DDKjitPCT	35.79	47.30	0.003	0.547	58.29	56.59	0.632	0.091
DDKjitABS	34.49	52.76	0.001	0.604	61.78	70.36	0.109	0.290
DDKjitREG	40.20	65.96	0.001	0.592	57.03	67.31	0.090	0.325
DDKsdi	3.56	4.05	0.029	0.410	4.52	4.16	0.252	0.250
DDKcvi	9.94	13.66	<0.001	0.707	12.55	13.12	0.633	0.102

The data was described using means, and a two-sample t-test was performed to compare the normal control group (HC) and the Parkinson's patient group (PD). Hedge's g was used as the effect size measure.

Supplementary Table 2:

	Cortical area	Cluster size (mm2)	Cluster-wise P value	MNI coordinates (mm)		
				x	y	z
ROI	fusiform	748.54	0.02350	30.7	-39.9	-18.7

Supplementary Table 3: AAL90 Atlas Brain Regions and Corresponding Abbreviations

**Regions**	**abbr.**
Precental gyrus	PreCG.L
Precental gyrus	PreCG.R
Superior frontal gyrus, dorsolateral	SFGdor.L
Superior frontal gyrus, dorsolateral	SFGdor.R
Superior frontal gyrus, orbital part	ORBsup.L
Superior frontal gyrus, orbital part	ORBsup.R
Middle frontal gyrus	MFG.L
Middle frontal gyrus	MFG.R
Middle frontal gyrus, orbital part	ORBmid.L
Middle frontal gyrus, orbital part	ORBmid.R
Inferior frontal gyrus, opercular part	IFGoperc.L
Inferior frontal gyrus, opercular part	IFGoperc.R
Inferior frontal gyrus, triangular part	IFGtriang.L
Inferior frontal gyrus, triangular part	IFGtriang.R
Inferior frontal gyrus, orbital part	ORBinf.L
Inferior frontal gyrus, orbital part	ORBinf.R
Rolandic operculum	ROL.L
Rolandic operculum	ROL.R
Supplementary motor area	SMA.L
Supplementary motor area	SMA.R
Olfactory cortex	OLF.L
Olfactory cortex	OLF.R
Superior frontal gyrus, medial	SFGmed.L
Superior frontal gyrus, medial	SFGmed.R
Superior frontal gyrus, medial orbital	ORBsupmed.L
Superior frontal gyrus, medial orbital	ORBsupmed.R
Gyrus rectus	REC.L
Gyrus rectus	REC.R
Insula	INS.L
Insula	INS.R
Anterior cingulate and paracingulate gyri	ACG.L
Anterior cingulate and paracingulate gyri	ACG.R
Median cingulate and paracingulate gyri	DCG.L
Median cingulate and paracingulate gyri	DCG.R
Posterior cingulate gyrus	PCG.L
Posterior cingulate gyrus	PCG.R
Hippocampus	HIP.L
Hippocampus	HIP.R
Parahippocampal gyrus	PHG.L
Parahippocampal gyrus	PHG.R

Amygdala	AMYG.L
Amygdala	AMYG.R
Calcarine fissure and surrounding cortex	CAL.L
Calcarine fissure and surrounding cortex	CAL.R
Cuneus	CUN.L
Cuneus	CUN.R
Lingual gyrus	LING.L
Lingual gyrus	LING.R
Superior occipital gyrus	SOG.L
Superior occipital gyrus	SOG.R
Middle occipital gyrus	MOG.L
Middle occipital gyrus	MOG.R
Inferior occipital gyrus	IOG.L
Inferior occipital gyrus	IOG.R
Fusiform gyrus	FFG.L
Fusiform gyrus	FFG.R
Postcentral gyrus	PoCG.L
Postcentral gyrus	PoCG.R
Superior parietal gyrus	SPG.L
Superior parietal gyrus	SPG.R
Inferior parietal, but supramarginal and angular gyri	IPL.L
Inferior parietal, but supramarginal and angular gyri	IPL.R
Supramarginal gyrus	SMG.L
Supramarginal gyrus	SMG.R
Angular gyrus	ANG.L
Angular gyrus	ANG.R
Precuneus	PCUN.L
Precuneus	PCUN.R
Paracentral lobule	PCL.L
Paracentral lobule	PCL.R
Caudate nucleus	CAU.L
Caudate nucleus	CAU.R
Lenticular nucleus, putamen	PUT.L
Lenticular nucleus, putamen	PUT.R
Lenticular nucleus, pallidum	PAL.L
Lenticular nucleus, pallidum	PAL.R
Thalamus	THA.L
Thalamus	THA.R
Heschl gyrus	HES.L
Heschl gyrus	HES.R
Superior temporal gyrus	STG.L
Superior temporal gyrus	STG.R
Temporal pole: superior temporal gyrus	TPOsup.L

Temporal pole: superior temporal gyrus	TPOsup.R
Middle temporal gyrus	MTG.L
Middle temporal gyrus	MTG.R
Temporal pole: middle temporal gyrus	TPOmid.L
Temporal pole: middle temporal gyrus	TPOmid.R
Inferior temporal gyrus	ITG.L
Inferior temporal gyrus	ITG.R

Supplementary Table 4: Functional connectivity of speech metrics

DPI			
Correlations Between		*p*	R
ORBmid.R	CAU.R	0.003	0.833
ORBinf.R	SFGmed.L	0.002	0.856
ORBsupmed.R	ANG.L	0.005	0.805
PUT.L	HES.R	0.003	-0.838

NSR			
Correlations Between		*p*	R
PreCG.R	SFGmed.R	0.003	0.829
SFGmed.L	PoCG.R	0.002	0.848
SFGmed.R	PoCG.R	0.003	0.833
FFG.R	PoCG.R	0.002	0.843
PCG.L	MTG.R	0.003	-0.832
PCG.R	MTG.R	0.003	-0.832

DDKR			
Correlations Between		*p*	R
ORBinf.L	ORBsupmed.L	0.003	-0.835
ORBmid.L	ORBsupmed.R	0.001	-0.869
ORBmid.L	REC.R	0.004	-0.822
CUN.R	FFG.L	0.003	0.832
SOG.R	PoCG.L	0.003	0.830
ORBmid.R	SMG.R	0.002	0.841
ORBinf.R	SMG.R	0.004	0.822
PHG.L	CAU.L	0.001	-0.883
FFG.L	CAU.L	0.002	-0.847

Supplementary Table 5: Functional connectivity of UPDRS

UPDRS_off			
Correlations Between		*p*	R
SFGdor.L	ORBsup.L	0.001	0.858
PUT.R	ORBsup.R	0.003	0.829
CAL.L	IFGoperc.R	0.001	0.880

PreCG.L	IFGtriang.L	0.005	0.805
ORBmid.R	ORBsupmed.L	0.001	0.868
IFGoperc.L	ORBsupmed.L	0.002	0.846
ACG.R	ORBsupmed.L	0.001	0.873
FFG.R	ORBsupmed.L	0.003	0.824
IFGoperc.L	REC.L	0.003	0.825
ORBinf.R	REC.L	0.002	0.856
ACG.R	REC.L	0.000	0.894
DCG.R	REC.L	0.000	0.914
INS.R	INS.L	0.004	-0.810
IFGtriang.L	ACG.L	0.000	0.902
ORBsupmed.L	DCG.L	0.001	0.893
FFG.R	DCG.L	0.000	0.900
IOG.L	DCG.R	0.002	0.856
IPL.L	PCG.L	0.002	0.843
PAL.L	HIP.R	0.001	0.867
IPL.R	PHG.L	0.005	0.808
PoCG.L	PHG.R	0.004	0.810
PoCG.R	PHG.R	0.002	0.843
PAL.R	AMYG.L	0.002	0.840
REC.L	AMYG.R	0.003	0.834
CAL.R	AMYG.R	0.002	0.839

Supplementary Figure 1:

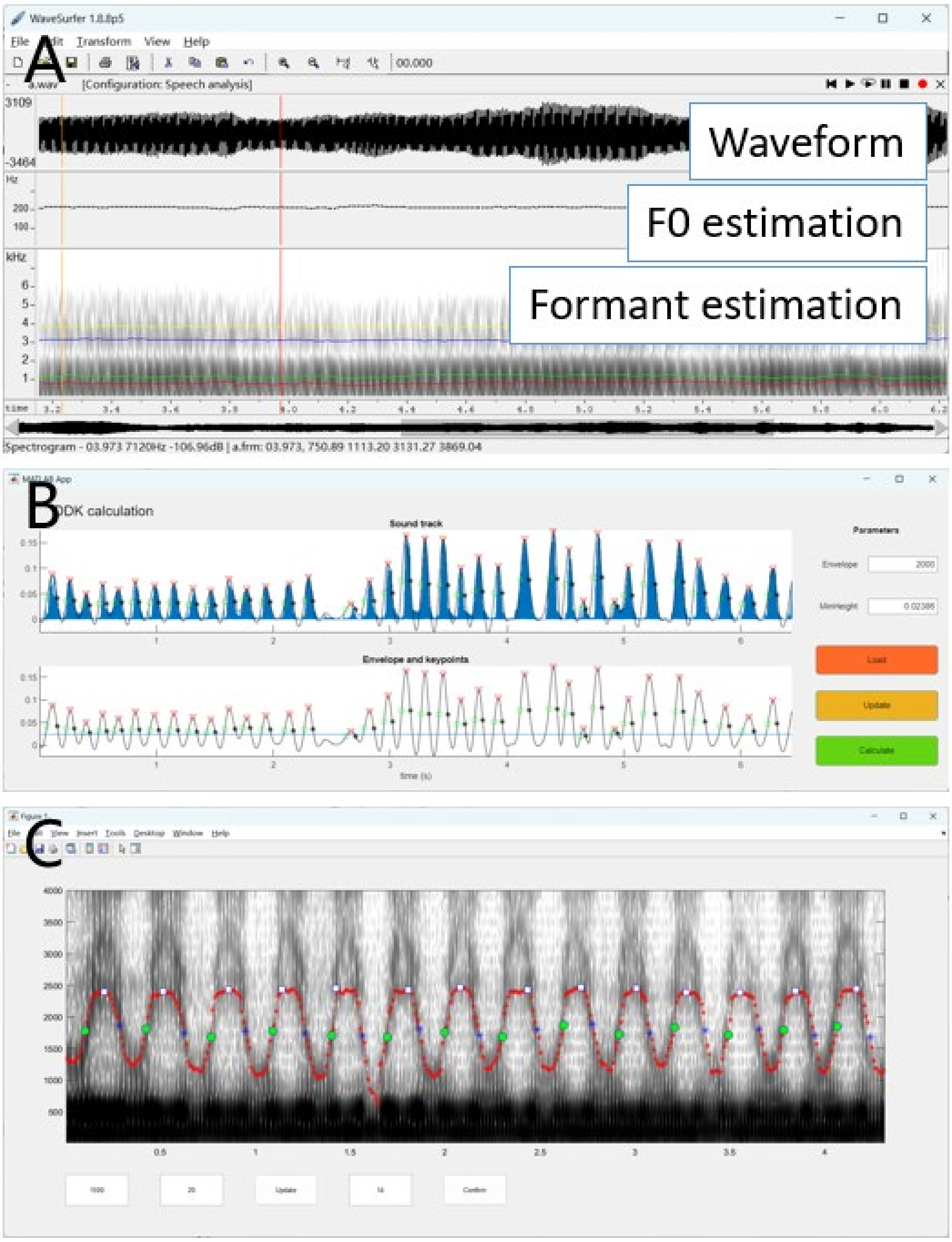

Supplementary Figure 2: Correlation between Seven Filtering Indicators Across Four Dimensions

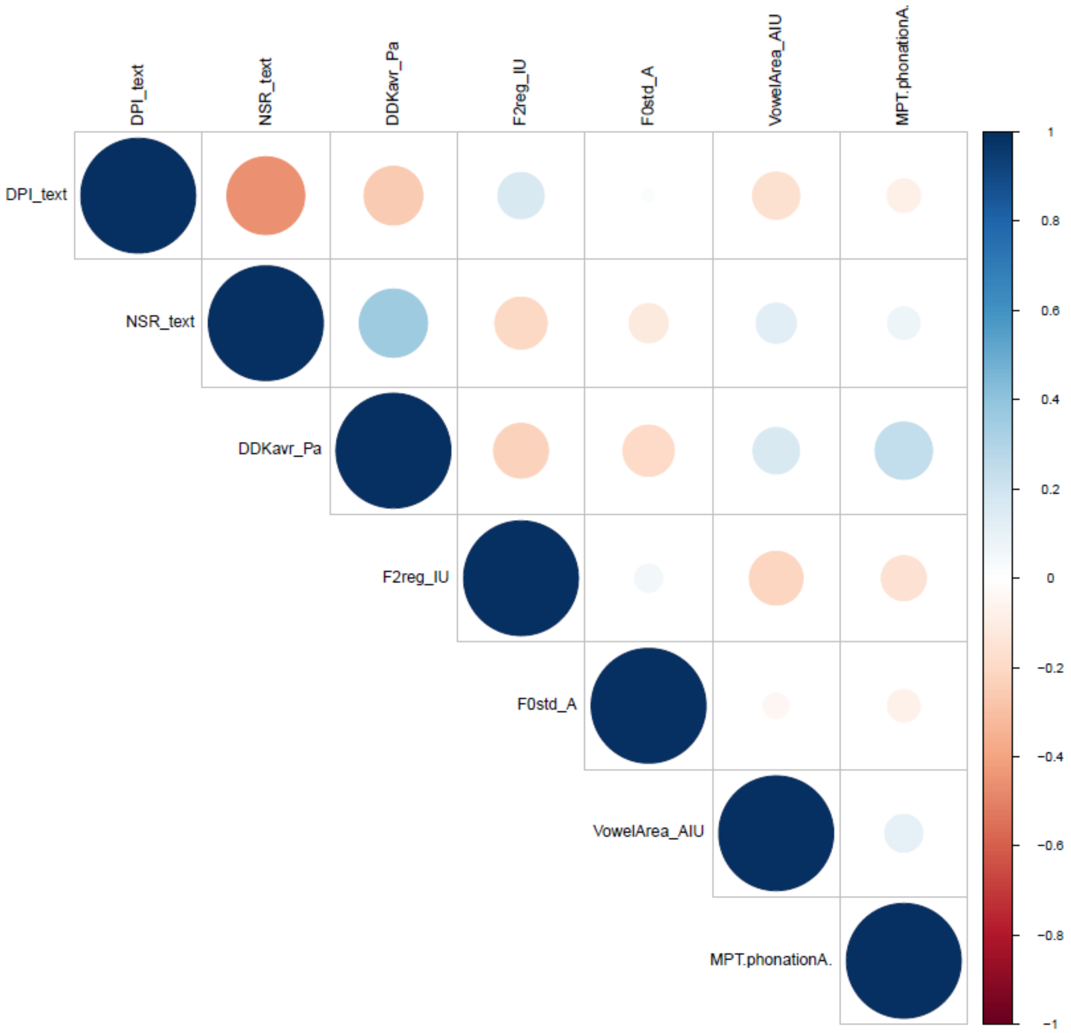

Correlation between Seven Filtering Indicators Across Four Dimensions: Respiration, Pronunciation, Phonation, and Prosody. All inter-indicator correlations are below 0.5.
