# Supplementary material for: Acoustic assessment in mandarin-speaking Parkinson’s disease patients and disease progression monitoring and brain impairment within the speech subsystem
Source: NPJ Parkinsons Dis. 2024 Jun 12;10:115. doi: 10.1038/s41531-024-00720-3 (PMC11169641; doi:10.1038/s41531-024-00720-3)
Supplement: Supplementary file 2 — reporting summary [file 41531_2024_720_MOESM2_ESM.pdf]

nature portfolio

Corresponding author(s): Jan Hlavnicka, Jianguo Zhang

Last updated by author(s): Apr 26, 2024

Reporting Summary

Nature Portfolio wishes to improve the reproducibility of the work that we publish. This form provides structure for consistency and transparency in reporting. For further information on Nature Portfolio policies, see our Editorial Policies and the Editorial Policy Checklist.

Statistics

For all statistical analyses, confirm that the following items are present in the figure legend, table legend, main text, or Methods section.

n/a	Confirmed	
☐	☒	The exact sample size (n) for each experimental group/condition, given as a discrete number and unit of measurement
☐	☒	A statement on whether measurements were taken from distinct samples or whether the same sample was measured repeatedly
☐	☒	The statistical test(s) used AND whether they are one- or two-sided<br>*Only common tests should be described solely by name; describe more complex techniques in the Methods section.*
☐	☒	A description of all covariates tested
☐	☒	A description of any assumptions or corrections, such as tests of normality and adjustment for multiple comparisons
☐	☒	A full description of the statistical parameters including central tendency (e.g. means) or other basic estimates (e.g. regression coefficient) AND variation (e.g. standard deviation) or associated estimates of uncertainty (e.g. confidence intervals)
☒	☐	For null hypothesis testing, the test statistic (e.g. F, t, r) with confidence intervals, effect sizes, degrees of freedom and P value noted<br>*Give P values as exact values whenever suitable.*
☒	☐	For Bayesian analysis, information on the choice of priors and Markov chain Monte Carlo settings
☒	☐	For hierarchical and complex designs, identification of the appropriate level for tests and full reporting of outcomes
☐	☒	Estimates of effect sizes (e.g. Cohen's d, Pearson's r), indicating how they were calculated

Our web collection on statistics for biologists contains articles on many of the points above.

Software and code

Policy information about availability of computer code

Data collection	N/a
Data analysis	WaveSurfer and MATLAB 2021b (MathWorks Inc., Natick, Massachusetts, USA)

For manuscripts utilizing custom algorithms or software that are central to the research but not yet described in published literature, software must be made available to editors and reviewers. We strongly encourage code deposition in a community repository (e.g. GitHub). See the Nature Portfolio guidelines for submitting code & software for further information.

Data

Policy information about availability of data

All manuscripts must include a data availability statement. This statement should provide the following information, where applicable:

- Accession codes, unique identifiers, or web links for publicly available datasets
- A description of any restrictions on data availability
- For clinical datasets or third party data, please ensure that the statement adheres to our policy

The data that support the findings of this study are available from the corresponding author upon request.

Research involving human participants, their data, or biological material

Policy information about studies with human participants or human data. See also policy information about sex, gender (identity/presentation), and sexual orientation and race, ethnicity and racism.

Reporting on sex and gender	Yes
Reporting on race, ethnicity, or other socially relevant groupings	N/a
Population characteristics	Yes
Recruitment	N/a
Ethics oversight	Yes

Note that full information on the approval of the study protocol must also be provided in the manuscript.

Field-specific reporting

Please select the one below that is the best fit for your research. If you are not sure, read the appropriate sections before making your selection.

☒ Life sciences ☐ Behavioural & social sciences ☐ Ecological, evolutionary & environmental sciences

For a reference copy of the document with all sections, see nature.com/documents/nr-reporting-summary-flat.pdf

Life sciences study design

All studies must disclose on these points even when the disclosure is negative.

Sample size	80 PD patients and 80 health control
Data exclusions	The inclusion criteria for PD cohort were: (1) the diagnosis of idiopathic PD and meeting the UK Brain Bank criteria for PD [57]; and with a disease duration of 5 years or more; (2) normal cognitive function (scores of Montreal Cognitive Assessment (MoCA) ≥24) or mild cognitive impairment (meeting the criteria for the diagnosis of PD-MCI level I [58] and 16?MoCA<24) [59]; (3) completing the entire experiment and cooperating fully with the investigators; (4) meeting the quality control standards on neuroimaging and speech examination.
Replication	Yes
Randomization	No randomization
Blinding	No

Reporting for specific materials, systems and methods

We require information from authors about some types of materials, experimental systems and methods used in many studies. Here, indicate whether each material, system or method listed is relevant to your study. If you are not sure if a list item applies to your research, read the appropriate section before selecting a response.

Materials & experimental systems

n/a	Involved in the study	
☒	☐	Antibodies
☒	☐	Eukaryotic cell lines
☒	☐	Palaeontology and archaeology
☒	☐	Animals and other organisms
☒	☐	Clinical data
☒	☐	Dual use research of concern
☒	☐	Plants

Methods

n/a	Involved in the study	
☒	☐	ChIP-seq
☒	☐	Flow cytometry
☐	☒	MRI-based neuroimaging

Plants

Seed stocks: No involved

Novel plant genotypes: No involved

Authentication: No involved

Magnetic resonance imaging

Experimental design

Design type: Structural and founctional MRI

Design specifications: Speech Examination

Behavioral performance measures: Scale scoring and speech features

Acquisition

Imaging type(s): 3DT1 BOLD

Field strength: 3.0

Sequence & imaging parameters: three-dimensional sagittal T1-weighted-3D magnetization-prepared rapid acquisition gradient echo (MPRAGE) sequence (repetition time, 6.6 ms; echo time, 3.1 ms; flip angle, 8°; matrix size, 240 × 240; isotropic voxel, 1 × 1 × 1 mm3; number of slices, 196)

Area of acquisition: whole brain

Diffusion MRI: ☐ Used ☒ Not used

Preprocessing

Preprocessing software: freesurfer, SPM12, GRETNA 2.0.0 toolbox

Normalization: To normalize the regional thickness in each patient, we normalized the regional cortical thickness by the global mean of cortical thickness at an individual level and subsequently, the results were z-score normalized to the healthy controls

Normalization template: MNI

Noise and artifact removal: no involved

Volume censoring: no involved

Statistical modeling & inference

Model type and settings: no involved

Effect(s) tested: no involved

Specify type of analysis: ☒ Whole brain ☐ ROI-based ☐ Both

Statistic type for inference (See Eklund et al. 2016): no involved

Correction: no involved

Models & analysis

n/a	Involved in the study
☐	☒ Functional and/or effective connectivity
☒	☐ Graph analysis
☒	☐ Multivariate modeling or predictive analysis

Functional and/or effective connectivity	The intrinsic connectivity network within the brain is comprised of nodes and edges. Utilizing the AAL90 atlas, the brain is segmented into 90 nodes, and a functional connectivity network (FCN) of size 90 * 90 is constructed by computing correlations of the time series for these 90 nodes.
